# Perceived Enablers of and Barriers to Serious Game as Oral Histology Learning Strategy for Undergraduate Dental Students

**DOI:** 10.3390/dj12090280

**Published:** 2024-08-31

**Authors:** Lisa R. Amir, Salsabila N. Dewatmoko, Irene C. Leonardy, Rezon Yanuar, Dewi F. Suniarti, Erik Idrus, Kawin Sipiyaruk, Ria Puspitawati, Yuniardini Wimardhani

**Affiliations:** 1 Department of Oral Biology, Faculty of Dentistry, Universitas Indonesia, Jakarta 10340, Indonesia; dewi.fatma@ui.ac.id (D.F.S.); erik.idrus31@ui.ac.id (E.I.); r_puspitawati@ui.ac.id (R.P.); 2 Dental Education Unit, Faculty of Dentistry, Universitas Indonesia, Jakarta 10340, Indonesia; 3 Dentistry Study Program, Faculty of Dentistry, Universitas Indonesia, Jakarta 10340, Indonesia; 4 Department of Pharmacology, School of Dentistry, Health Sciences University of Hokkaido, Hokkaido 061-0293, Japan; reyson.yanuar@gmail.com; 5 Department of Orthodontics, Faculty of Dentistry, Mahidol University, Bangkok 10400, Thailand; kawin.sip@mahidol.ac.th; 6 Department of Oral Medicine, Faculty of Dentistry, Universitas Indonesia, Jakarta 10340, Indonesia; yuniardini@ui.ac.id

**Keywords:** serious game, complementary learning, enabler factors, barrier factors, innovative learning

## Abstract

Previously, we reported the serious game *HistoRM* as an innovative learning approach for an oral histology course. This study aimed to examine the impact of *HistoRM* on summative assessment and the enablers and barriers factors affecting *HistoRM* as an interactive learning strategy in an oral histology course. A crossover randomized controlled trial was performed. Study participants were first-year dental students at the Universitas Indonesia. The average final grades of students who participated in *HistoRM* serious game were significantly higher than those of students who did not participate in this trial (*p* < 0.001). Positive responses of *HistoRM* were observed in the learning content, games and learning experience domains. The enabler factors most recognized by the students were the game method, which helped students to understand the learning materials; the immediate feedback provided following each gameplay; as well as the fun and interesting gameplay. The barrier factors most recognized by the students were some challenges of the gameplay, which resulted in a longer time needed to study. While the *HistoRM* serious game can stimulate student motivation and engagement in learning oral histology, overcoming the barriers is essential for the implementation of serious games as a complementary learning approach in the dental curriculum.

## 1. Introduction

The integration of technology into education undoubtedly accelerated by the COVID-19 pandemic has caused a fundamental shift in pedagogical strategies [1,2,3]. The necessity for digital transformation in higher education is in line with the introduction of innovative e-learning applications such as games with pedagogical purposes known as serious games [4,5,6,7,8,9,10]. Games are appealing because they accomplish a goal that differs from conventional teaching, providing students with engaging problems and providing potential methods to address problems rather than emphasizing information transmission and memory as in a traditional lecture-based approach [6,7]. This provides opportunities for students to develop higher-order thinking such as application and analysis. A learning strategy that incorporates serious games is a promising and innovative learning approach in dentistry. The preference for video games has led educators to integrate games in an educational context. Knowledge acquisition, student learning motivation and satisfaction are positively influenced by a game-based approach [11,12,13,14].

We recently reported a novel teaching approach for asynchronous, interactive dental education environments, namely, *HistoRM*, a serious game for an oral histology course [4]. This new learning strategy was introduced to overcome difficulties in understanding oral histology-related subjects in the Universitas Indonesia dentistry study program. This study’s findings demonstrated that oral histology learning with *HistoRM* improved students’ understanding of the subject matter. The *HistoRM* game was well received by students as a way to improve their comprehension of the microscopic structures of oral tissues. However, it is unclear what the factors are that influence students’ acceptance of these new learning methods. Therefore, in this present study, we aimed to further analyze the enablers of and barriers to complementary learning of *HistoRM* for an oral histology course. In addition, the impact of *HistoRM* on summative tests for short-term knowledge retention was evaluated. Understanding the enablers of and barriers to the *HistoRM* serious game will support the improvement of the game.

## 2. Materials and Methods

### 2.1. Design and Study Participants

This crossover randomized controlled trial study was approved by Universitas Indonesia Dental Research Ethics Committee (87/ethical approval/FKGUI/X/2022) and registered with the ISRCTN Registry Service (registration number ISRCTN11006820). Undergraduate first-year dental students of the 2022/2023 academic year were invited to participate in the study. Students who signed the informed consent form were included in the study. Pre-tests were performed prior to the study. The pre-test and GPA scores were taken into consideration for random allocation of the participating students to minimize bias between groups. The students were divided into two groups: group 1 studied the *HistoRM* for 3 days, performed post-test 1 and were given a practicum hand-out (script-based) to study for another four days. Post-test 2 was carried out 7 days after the intervention. Group 2 studied the practicum hand-out (script-based) in the first three days, followed by *HistoRM*. Two learning materials of microscopic structures of oral mucosa (topic 1) and tooth and periodontal tissues (topic 2) were designed with complementary learning of *HistoRM*. The experimental study was repeated for these two topics, representing the cross-over design. The short-term knowledge retention was examined during the summative mid-term test and final-term test, conducted approximately one-month post-intervention, utilizing similar questions for assessing the learning outcomes of the subject matters. Student participation in this study was voluntary, with a random allocation ratio 1:1. The summative scores of non-participating students were subsequently compared with those of participating students. The average summative scores were extracted specifically for the scores of oral histology learning topics. The reporting of this study followed the Consolidated Standards of Reporting Trials (CONSORT) guidelines [4,15].

### 2.2. HistoRM Game

**Figure 1:**
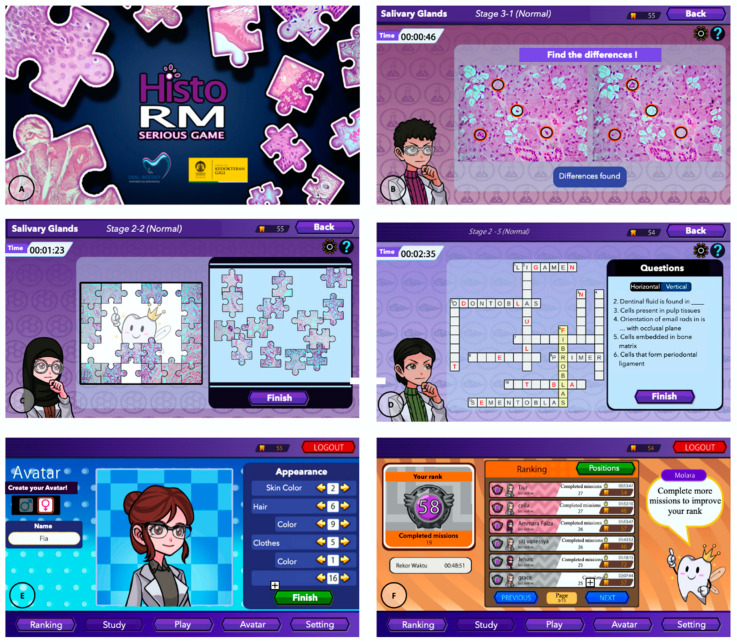
The *HistoRM* game was incorporated in the Sharable Content Object Reference Model (SCORM) activity in the Universitas Indonesia Moodle-based learning management system (EMAS) (**A**). Finding differences (**B**); matching pictures (**C**); crosswords (**D**); entertaining features of player avatars were designed as part of the fun elements of *HistoRM* (**E**); student achievement and number of rewards were shown in the leaderboard (**F**).

The Unity game platform was used to develop a serious game called *HistoRM* as previously described [4]. *HistoRM* was incorporated into the Sharable Content Object Reference Model (SCORM) activity in the Universitas Indonesia Moodle-based learning management system (EMAS). *HistoRM* consisted of four kinds of gameplay: (1) matching pictures; (2) finding differences, (3) jigsaw puzzles, and (4) crossword puzzles. There were three stage levels for every topic, with five missions in each level, making for a total of fifteen missions for every topic. There were two sessions for each mission: a puzzle game session and a quiz session. The quiz session can be taken upon completion of the puzzle game session, and it could be repeated as many times as needed. No time limit for playing *HistoRM* was applied. Entertaining features were added to the *HistoRM* game, such as player avatars created specifically for each participant, a personalized background, and music. Avatar customization options included clothing, accessories, hair color, and style. The avatar’s facial expression varied according to how they interacted with the games. A character with the appearance of a human tooth, named Molara, was created to guide the students. Molara began by outlining the learning objectives for each step and providing guidance on how to play the game, a hint feature to help students when they were having trouble finishing the puzzle game and giving prompt feedback with an explanation regarding the quiz topics (Figure 1A–F).

### 2.3. Questionnaire

The evaluation of student perceptions of *HistoRM* was conducted with a questionnaire consisting of five questions concerning the students’ perception of the *HistoRM* game and three open-ended questions related to the enablers and barrier factors to the *HistoRM* game, as well as their recommendations for improvement. The enablers and barrier factors were classified into learning education and game and learning experience aspects.

### 2.4. Statistical Analysis

Data normality was tested with the Shapiro–Wilk test. The fold differences of scores following intervention and the impact of *HistoRM* game on final grades of participating students in comparison with the scores of non-participating students were analyzed with a Kruskal–Wallis and independent *t*-test, respectively. The correlation between final grades and the number of completed missions, the duration of playing *HistoRM* and game score was tested with Pearson correlation analysis. Statistical analysis was performed using the SPSS version 25.0 (IBM Corp., Armonk, NY, USA). The level of statistical significance was accepted at 0.05.

## 3. Results

Seventy-four first-year students (53.6%) participated in the studies, and 85.1% of them were female, which reflected the majority of the undergraduate dental students. Almost all participants filled out the questionnaire (98.6%).

### 3.1. Impact of HistoRM on Knowledge Retention

**Figure 2:**
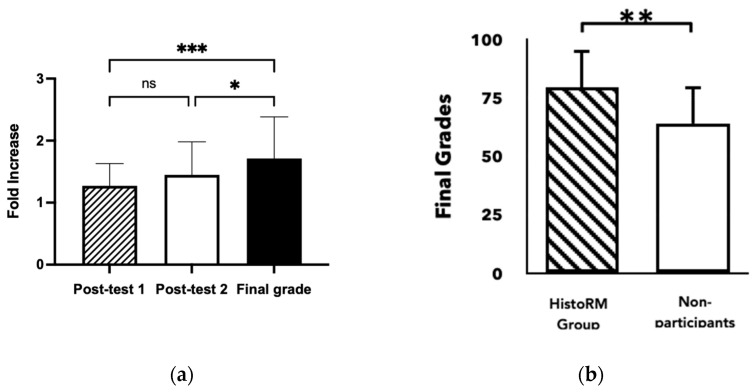
Impact of *HistoRM* on summative tests: (**a**) fold increase knowledge gain score compared to pre-test; (**b**) grades of summative test of *HistoRM* group and non-participants. (* *p* < 0.05, ** *p* < 0.05, *** *p* < 0.05).

**Figure 3:**
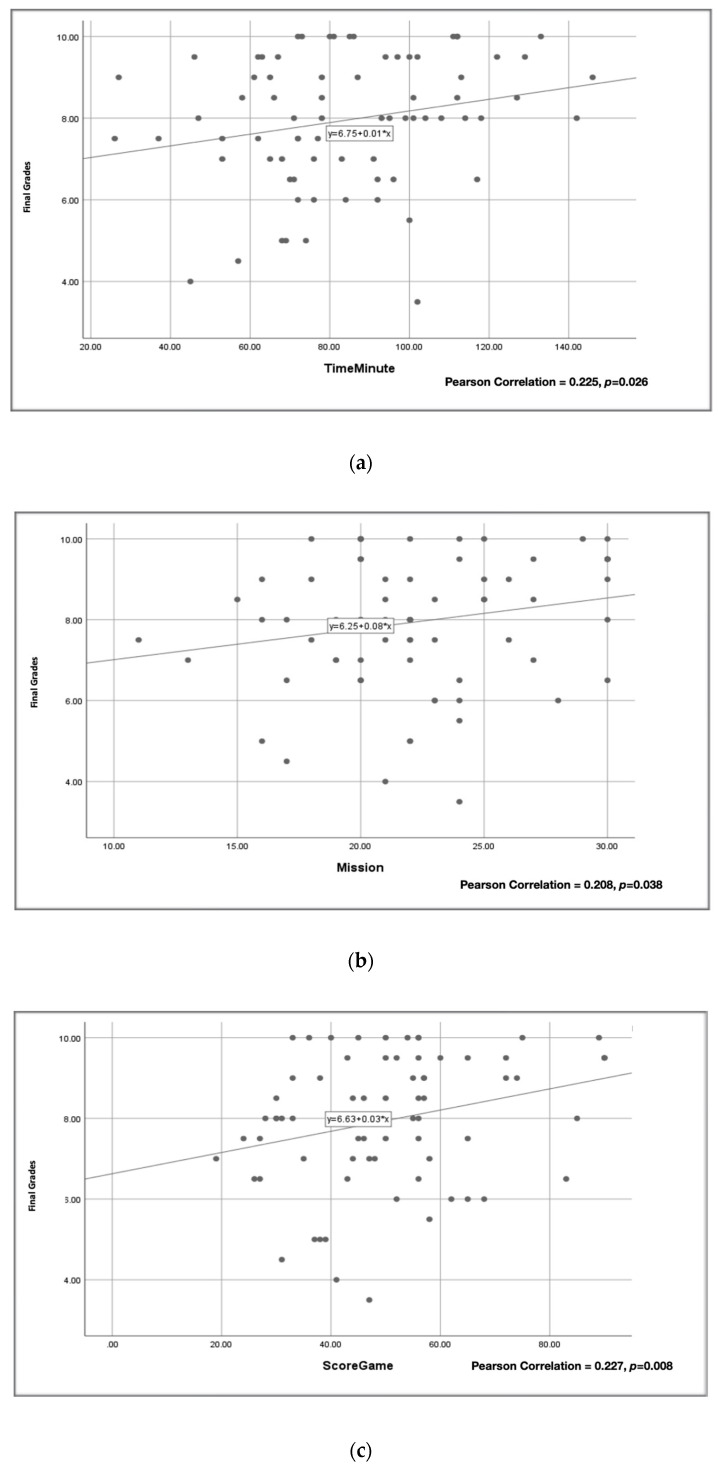
The correlation of *HistoRM* game characteristics with students’ understanding of the subject matter: (**a**) correlation of duration of *HistoRM* play time and students’ summative scores; (**b**) correlation of number of missions of *HistoRM* and students’ summative scores; (**c**) correlation of the *HistoRM* score with students’ summative scores.

Previously, we showed that *HistoRM* affected students’ cognitive skills, evaluated in post-tests observed on day 3 and day 7 after *HistoRM* game intervention. Further, we analyzed the knowledge retention, evaluated during the final examination (summative test) of the oral histology course. This study demonstrated an additional increase in cognitive skills one month after *HistoRM* game intervention was implemented. The fold increase of the pre-test score was statistically significant compared to the day 7 post-test score (*p* < 0.001, Kruskal–Wallis test) (Figure 2a). We observed the average final grades of the students who participated in this serious game study (79.52 ± 1.6) and found the grades were significantly higher than those of non-participating students (64.03 ± 2.05) (*p* < 0.001, independent *t*-test), indicating the effectiveness of *HistoRM* as a complementary learning method (Figure 2b). The students’ summative scores were significantly correlated with the number of completed missions, the duration of playing HistoRM and game score (Pearson correlation analysis) (Figure 3).

### 3.2. Impact of HistoRM Game on Learning Motivation

Face validity, Cronbach’s alpha, and Intraclass Correlation Coefficient (ICC) were performed to test the validity and reliability of the questionnaire. The Cronbach alpha score was 0.85 and it can be classified as reliable. The ICC score had an excellent agreement (r = 0.98, 95% Confidence Interval (CI), 0.94–0.99).

The students’ perception of the HistoRM game as an oral histology learning approach was positive. More than 90% of the participants responded that *HistoRM* gave students motivation to learn and they agreed that this new learning strategy could be developed as a complementary learning method for other learning subjects. In total, 86.4% of the participants agreed that *HistoRM* provided greater learning motivation than script-based handouts, although only 79.5% of the students indicated that they were keen to play games in their free time (Table 1).

### 3.3. Enablers of and Barriers to HistoRM as a Learning Strategy for Oral Histology

The enabler and barrier factors affecting *HistoRM* as a complementary learning strategy for oral histology can be classified as education and game and learning experience aspects (Table 2 and Table 3). The enabler factors most recognized by the students were the game method, which helped students to understand the learning materials; the immediate feedback provided after each game; and the fun and interesting gameplay. The barrier factors most recognized by the students were some of the games being difficult, causing them to take them longer to study or to complete the game mission, and some of the questions in the quiz being too difficult with not enough hints.

The suggestions most recommended by the participants were: [1] implementing the serious game for other learning subjects, [2] reducing the difficulty of gameplay by providing more hints, [3] improving access to *HistoRM* via mobile phones, [4] providing a review option upon the completion of the gameplay in each mission; and [5] unlimited access to *HistoRM* for self-directed learning.

In this study, student engagement was analyzed using *HistoRM* analytic reports and the students’ narrative comments on their perception of the course evaluation. Some of the students’ comments on the enabler factors were as follows:

*“..The Find The Difference game is really fun and the explanation of each question is really good. it’s short and easy to understand..”*

*“..When putting together a puzzle & finding the difference, I will indirectly look at the image for a long time, making it easier for me to become familiar with the image. The feedback and questions after the puzzle also really helped me remember the name, function and characteristics of each structure..”*

*“..HistoRM is very interesting and honestly very useful. Moreover, there is a pop quiz every time we finish completing a puzzle, which really adds insight. This is a method of learning and recalling histology material that is super fun and doesn’t make you feel bored when studying it. The avatar is also cute, we can customize it however we like! This HistoRM really had a real impact on my understanding of oral histology..”*

Other students commented on barrier factors affecting learning oral histology with *HistoRM*, as follows:

*“..Some of the missions were too difficult. For example, find the difference gameplay, if the structures asked were too detailed. But I understand the purpose is to help us understand oral histology and be successful in the summative test..”*

*”..There was no overview of the results after we finished the mission and the overall games..”*

*”..Some of the difficult missions took longer time to complete and it is recommended to add more hints to help students..”*

*”..Compare to studying with script-based, it took longer time to study with HistoRM. We have assignments of other courses, it was therefore harder to complete the game within the designated time frame. It is recommended to set more flexible time to study oral histology with HistoRM..”*

*”..It is not possible to proceed to the quiz before completing the puzzle..”*

## 4. Discussion

This present study was conducted to evaluate the impact of serious game *HistoRM* on the summative assessment of undergraduate dental students and the enablers and barriers affecting *HistoRM*. Understanding enabler and barrier factors in learning oral histology with HistoRM is essential for continued refinement and replication of the course. The results of this study indicated a positive impact of *HistoRM* as a complementary learning tool on short-term knowledge retention of oral histology. The enabler factors included fun and interesting gameplay and the fact that the immediate feedback helped dental students understand the learning materials. The barrier factors affecting *HistoRM* were difficult gameplay in some of the missions, which therefore required a longer time to study and complete the game. This present study presents evidence of serious games as a promising and innovative learning strategy in dentistry. Active engagement can help develop critical thinking skills in adults [16]. Knowledge acquisition, students’ learning motivation and students’ satisfaction are positively influenced by a game-based approach [4,5,6,7,8,9,10].

Student engagement is defined as a student’s willingness to actively participate in the learning process, as well as showing continuous involvement with a positive emotional condition towards the learning experience, and is important in teaching and learning activities. Student engagement in learning through serious games is a requisite to achieve the maximum result [17]. Based on the result of students’ perspectives, it is found that *HistoRM* motivates them to learn, of which the aspect of cognitive engagement is more dominant than other aspects. Serious games should be fun to play, but on the other hand, they possess learning benefits that can easily be achieved [18].

Confounding factors in this study included the length of time that the students played *HistoRM*, the study load of other courses and non-academic activities. Students who could not achieve the minimum mission target within the specified time limit were excluded from this study. High study load and short duration of studying *HistoRM* were among the main barrier factors. Previously, evidence suggested that subject matter learned over a longer duration results in superior long-term knowledge retention to that of subject matter learned over a shorter duration [19,20,21,22,23]. Spaced or distributed learning was described in terms of increased time spent on delivering courses and increased number of recall episodes. Retention of knowledge of basic sciences tends to deteriorate throughout the extent of an average medical course as clinical medicine predominates the courses of the later years of the medical curriculum [24]. A similar phenomenon is observed in the dentistry curriculum. During Indonesian national competence exams, the grades of basic dental science subjects tend to be lower compared to clinical dental science subjects (unpublished data). It is therefore imperative to evaluate the implementation of *HistoRM* to overcome the barrier factors. Repetition of a game cycle can lead to the accomplishment of the expected learning outcomes. Optimal long-term retention of knowledge can be achieved, including active recall and expanding repetition intervals [25].

This present study presents evidence for the future development and improvement of a serious game. However, this study had some limitations, including the short intervention time and the fact that the number of study participants only consisted of 74 (53.6%) first-year pre-clinical dental students, as participation was voluntary. Previous studies showed low knowledge retention of basic science in the medical curriculum over the span of medical education [22]. As this present study assessed short-term knowledge acquisition, the impact of *HistoRM* on long-term knowledge retention of oral histology requires further study.

## 5. Conclusions

This present study highlights the importance of introducing an innovative learning approach to enhance knowledge acquisition and improve the overall learning environment. The serious game *HistoRM* stimulates dental student motivation and engagement in learning oral histology. Overcoming the barriers to *HistoRM* is essential for its implementation as a complementary learning strategy in the dental curriculum.

## 6. Patents

Patent application for the *HistoRM* game is currently in preparation

## Figures and Tables

**Table 1:** Student perception of *HistoRM* for oral histology learning methods.

*Statements*	Strongly Disagree	Disagree	Agree	Strongly Agree
1. *HistoRM* serious game motivated me to learn	1 (1.4%)	5 (7%)	41 (56%)	26 (35.6%)
2. *HistoRM* serious game motivated me to learn more than script-based hand-out	1 (1.4%)	9 (12.2%)	28 (37.8%)	36 (48.6%)
3. *HistoRM* serious game stimulated a new learning strategy	0 (0%)	1 (1.4%)	36 (48.6%)	37 (50%)
4. *HistoRM* serious game created an interesting learning method	1 (1.4%)	1 (1.4%)	38 (51%)	34 (46.2%)
5. *HistoRM* serious game could be developed for other learning topics as a complementary method	1 (1.4%)	3 (4.8%)	35 (47.6%)	34 (46.2%)

**Table 2:** Enabler factors that influence students’ positive preference toward *HistoRM* as a learning strategy for oral histology.

Enablers	Number of Participants	%	Mean of Final Grades
**1. Education Aspect**			**81.6 ± 13.4**
1.1 Direct feedback	18	14.63%	
1.2 Questions after each gameplay	10	8.13%	
**1.3 Clear explanation of learning content**	10	8.13%	
Subtotal		**30.89%**	
**2. Game Aspect**			**78.9 ± 15.2**
2.1 Gameplay	18	14.63%	
2.2 Game characters	7	5.69%	
2.3 Game visuals	9	7.32%	
Subtotal		**27.64%**	
**3. Learning Experience Aspect**			**79.4 ± 16.6**
3.1 Fun learning method	17	13.82%	
3.2 The method helps students understand the learning content	21	17.07%	
3.3 Motivates students to learn	4	3.25%	
3.4 The method helps students remember/recall learning content	9	7.32%	
Subtotal		**41.46%**	
**Total**		**100.00%**	
**Number of aspect preffered**			
1. Education aspect	9	12.16%	
2. Game aspect	8	10.81%	
3. Learning experience aspect	15	20.27%	
4. Education and game aspect	2	2.70%	
5. Education and learning experience aspect	11	14.86%	
6. Game and learning experience aspect	13	17.57%	
7. All three aspects	15	20.27%	

**Table 3:** Barrier factors that influence students’ positive preference for *HistoRM* as a learning strategy for oral histology.

Barriers	Number of Participants	%	Mean of Final Grades
**1. Education Aspect**			**85.0 ± 15.41**
1.1 Some questions are difficult	10	12.66%	
1.2 Some questions need additional hints	7	8.86%	
Subtotal			
**2. Game Aspect**			**80.5 ± 15.71**
2.1 Some gameplay is difficult	28	35.44%	
2.2 Some gameplay needs additional hints	7	8.86%	
2.3. Sometimes the images are not visible (bugs)	6	7.59%	
Subtotal			
**3. Learning Experience Aspect**			**78.0 ± 15.39**
3.1 Longer study time	17	21.52%	
3.2 Access to *HistoRM* game limited to desktop/laptop	2	2.53%	
3.3 Limited time to play	2	2.53%	
Subtotal			
**Total**			
**Number of aspects preferred**			
1. Education aspect	0	0.00%	
2. Game aspect	10	13.70%	
3. Learning experience aspect	4	5.48%	
4. Education and game aspect	1	1.37%	
5. Education and learning education aspect	4	5.48%	
6. Game and learning education aspect	33	45.21%	
7. No negative preferences	17	23.29%	

## Data Availability

Data are available upon reasonable request.
